# Chronic experimental autoimmune encephalomyelitis is an excellent model to study neuroaxonal degeneration in multiple sclerosis

**DOI:** 10.3389/fnmol.2022.1024058

**Published:** 2022-10-19

**Authors:** Rhonda R. Voskuhl, Allan MacKenzie-Graham

**Affiliations:** UCLA MS Program, Department of Neurology, David Geffen School of Medicine, University of California, Los Angeles, Los Angeles, CA, United States

**Keywords:** atrophy, cerebral cortex, demyelination, inflammation, neurodegeneration, multiple sclerosis, experimental autoimmune encephalomyelitis

## Abstract

Animal models of multiple sclerosis (MS), specifically experimental autoimmune encephalomyelitis (EAE), have been used extensively to develop anti-inflammatory treatments. However, the similarity between MS and one particular EAE model does not end at inflammation. MS and chronic EAE induced in C57BL/6 mice using myelin oligodendrocyte glycoprotein (MOG) peptide 35–55 share many neuropathologies. Beyond both having white matter lesions in spinal cord, both also have widespread neuropathology in the cerebral cortex, hippocampus, thalamus, striatum, cerebellum, and retina/optic nerve. In this review, we compare neuropathologies in each of these structures in MS with chronic EAE in C57BL/6 mice, and find evidence that this EAE model is well suited to study neuroaxonal degeneration in MS.

## Introduction

Multiple sclerosis (MS) is an autoimmune and neurodegenerative disease of the central nervous system (CNS). Axonal degeneration, synaptic and neuronal loss, and demyelination in the context of inflammation are characteristic of the disease (Trapp et al., 1998, 1999; Peterson et al., 2001; Stadelmann et al., 2011; Friese et al., 2014; Mock et al., 2021). Over time these processes result in atrophy that can be visualized by magnetic resonance imaging (MRI) (Miller et al., 2002; Zivadinov and Bakshi, 2004; Hulst and Geurts, 2011). Brain parenchymal fraction atrophy (Rudick et al., 1999; Fisher et al., 2002) and whole brain gray matter (GM) atrophy (Fisher et al., 2008) have been documented in both relapsing-remitting MS (RRMS) (Chard et al., 2002) and secondary progressive MS (SPMS) (Larochelle et al., 2016). Further, GM atrophy of neuroanatomical structures has been associated with specific disabilities (Bakshi et al., 2001; Calabrese et al., 2009, 2010; MacKenzie-Graham et al., 2016) and cumulative disease progression (Simon, 2001). Thus, GM substructure atrophy and neurodegenerative processes therein are highly relevant to disability progression in MS (Bermel and Bakshi, 2006).

Inflammation plays a critical role in MS; however, it is not clear how inflammation causes neurodegeneration. Correlations between early T2-lesion loads and subsequent atrophy (Chard et al., 2003), as well as gadolinium-enhancing lesions and brain atrophy have been shown (Lin and Blumhardt, 2001) and there is clearly a neuropathologic relationship between inflammation and axonal transection in RRMS (Trapp et al., 1998). There is also a relationship between inflammation and both demyelination and neurodegeneration in progressive MS (Frischer et al., 2009), albeit not as strongly as in RRMS. Anti-inflammatory disease-modifying treatments (DMTs) reduce relapses and have robust immunomodulatory effects, but there is still an unmet need for direct neuroprotective treatments that target cells within the CNS. Understanding mechanisms that drive neurodegeneration are crucial to identifying targets for therapeutic interventions to halt and repair disabilities.

**FIGURE 1:**
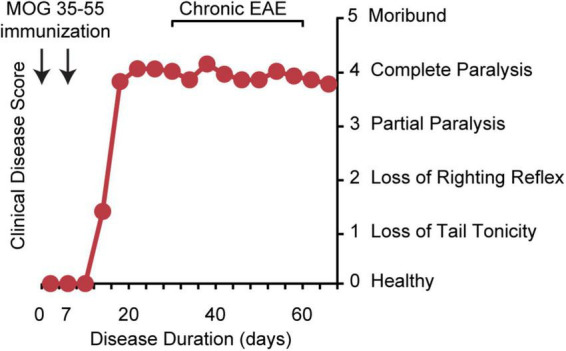
Chronic EAE disease induction and clinical disease course. Chronic EAE is induced by subcutaneous immunization with MOG peptide 35–55 and mycobacterium tuberculosis in complete Freund’s adjuvant at day 0 and again at day 7. Pertussis toxin is injected intraperitoneally on day 0 and day 2. Clinical disease is scored from 0 to 5 with 0 being healthy, 1 complete loss of tail tonicity, 2 loss of righting reflex, 3 partial paralysis of a limb, 4 complete paralysis of one or both hind limbs, and 5 moribund. Clinical signs of disease are usually first observed between days 10 and 15, followed by the chronic phase of disease, characterized by severe walking disability and progressive gray matter atrophy.

There is no perfect model for MS. The most appropriate model should be chosen based on the question asked. Experimental autoimmune encephalomyelitis (EAE) has been widely used as an animal model for immune studies in MS (Steinman and Zamvil, 2005; Ransohoff, 2012) and almost all DMTs have been tested in the EAE model (Denic et al., 2011; Ransohoff, 2012). MS has multiple presentations, from relapsing to secondary progressive to primary progressive MS. There are multiple presentations of EAE as well, from monophasic disease in the B10.PL mouse immunized with myelin basic protein (MBP) peptide Ac1-9 (Ando et al., 1989; Pastor et al., 2009) to relapsing-remitting disease in the SJL mouse immunized with proteolipid protein (PLP) peptide 139–151 (Brocke et al., 1996; Voskuhl, 1996) to chronic disease in the C57BL6 mouse immunized with myelin oligodendrocyte glycoprotein (MOG) peptide 35–55 (Hjelmstrom et al., 1998; Meyer et al., 2020; Figure 1). As we will review, chronic EAE induced using MOG 35–55 peptide in the C57BL/6J mouse strain (MOG 35–55 EAE) shares many neuropathologies with MS.

It is believed that MS is caused by the autoimmune activation of peripheral T cells that then migrate to the CNS and initiate disease (Baecher-Allan et al., 2018). In EAE, disease is induced by immunization with CNS antigens to produce autoimmune activation (Voskuhl, 1996; Hjelmstrom et al., 1998) or by the adoptive transfer of T-cell clones specific to myelin proteins that infiltrate the CNS (Zamvil S. et al., 1985; Zamvil S. S. et al., 1985). Once in the CNS, immune cells lead to classic inflammatory WM lesions, consisting of T-cells and macrophages with activation of resident glia (astrocytes and microglia), in both MS and EAE (Lassmann, 1998; Lassmann and Ransohoff, 2004; Wu et al., 2008; MacKenzie-Graham et al., 2009). Axonal damage characterized by axonal ovoids and end bulbs has been shown in both chronic EAE in C57BL/6 mice (Spence et al., 2014; Meyer et al., 2020) and in MS (Trapp et al., 1998, 1999). Further, chronic EAE also exhibits WM hyperintensities on T2-weighted MRI (MacKenzie-Graham et al., 2006) and axonal damage has been demonstrated using diffusion tensor imaging (DTI) (Budde et al., 2008, 2009), again as in MS (Klawiter, 2013). These observations suggest that EAE is a good model to study WM lesions and axonal loss in MS.

Beyond these WM lesions, there are also changes in normal-appearing white matter (NAWM). DTI and magnetization transfer ratio (MTR) abnormalities are common in MS (Moll et al., 2011; Elliott et al., 2021). DTI abnormalities in NAWM (Ahrens et al., 1998; Sun et al., 2007; MacKenzie-Graham et al., 2012a; Aharoni et al., 2013) and decreased MTR in NAWM have also been reported in EAE (Aharoni et al., 2013). NAWM in MS is associated with axonal pathology, activated astrocytes and microglia (Moll et al., 2011; Lassmann et al., 2012), all elements that are observed in NAWM in the corpus callosum and spinal cord in EAE (Wu et al., 2008; Crawford et al., 2010; Mangiardi et al., 2011).

Here we will focus on GM pathologies, comparing chronic EAE in C57BL/6 mice to that which occurs in MS. These pathologies include astrocyte and microglia activation, demyelination, axonal loss, synaptic loss, and neuronal loss in the cerebral cortex, hippocampus, thalamus, striatum, cerebellum, retina/optic nerve, and corpus callosum. Based on this review, we conclude that chronic EAE is an excellent model to study GM atrophy and neuroaxonal degeneration in MS.

## Cerebral cortex

Atrophy of cerebral cortex was recognized almost two decades ago (De Stefano et al., 2003; Calabrese et al., 2007). Interestingly, cerebral cortex atrophy and clinical disability are consistently associated (Amato et al., 2004; Charil et al., 2007; Rocca et al., 2021). It has been recently demonstrated that there is a direct correlation between regional cerebral cortex atrophy and specific disabilities (MacKenzie-Graham et al., 2016). Cerebral cortex atrophy in MOG 35–55 induced chronic EAE in C57BL/6 mice was first demonstrated in 2012 (MacKenzie-Graham et al., 2012b) and has been validated multiple times since then (Itoh et al., 2017; Hamilton et al., 2019; Meyer et al., 2020). Landmark MS studies demonstrated axonal damage and transection in lesions in subcortical WM (Ferguson et al., 1997; Trapp et al., 1998) and subsequent analyses demonstrated transected neurites and apoptotic neurons in the cerebral cortex (Kidd et al., 1999; Peterson et al., 2001), cementing the importance of cortical pathology in MS. Axonal loss (using B3-tubulin and NF200 staining) in the cerebral cortex has been demonstrated in chronic EAE (MacKenzie-Graham et al., 2012b). Decreased neuronal density has been shown in the cerebral cortices of MS patients (Vercellino et al., 2005, 2009; Bevan et al., 2018) and pyramidal neuronal loss (annexin V, encephalopsin, NeuN, and Thy1-YFP) has also been shown multiple times in chronic EAE (Mangiardi et al., 2011; MacKenzie-Graham et al., 2012b; Burns et al., 2014; Stanojlovic et al., 2016), highlighting that cortical pathology is important in this MS model as well. A systematic study of cortical demyelination performed by Bo et al. (2003) observed extensive myelin loss and these findings have been validated (Lucchinetti et al., 2011; Kutzelnigg and Lassmann, 2014; Bevan et al., 2018). Small areas of cortical demyelination have been reported many times in chronic EAE (Errede et al., 2012; MacKenzie-Graham et al., 2012b; Burns et al., 2014). Furthermore, microglial and astrocyte activation have been shown in the cerebral cortices of MS patients (Vercellino et al., 2009), as has synaptic loss (Mock et al., 2021). Diffuse astrocyte and microglia activation in cerebral cortex also occurs in chronic EAE (Errede et al., 2012; Burns et al., 2014; Meyer et al., 2020), as well as synaptic loss (Burns et al., 2014; Itoh et al., 2017; Meyer et al., 2020). We summarize cerebral cortex pathologies in chronic EAE in Table 1.

**TABLE 1:** Pathologies in the cerebral cortex in EAE.

Pathology	References
**Cerebral Cortex**	
Astrocyte activation	Errede et al., 2012; Ravelli et al., 2019
Atrophy	MacKenzie-Graham et al., 2012b; Spence et al., 2014; Itoh et al., 2017; Hamilton et al., 2019; Meyer et al., 2020
Axonal damage	MacKenzie-Graham et al., 2012b
Demyelination	Girolamo et al., 2011; Mangiardi et al., 2011; Errede et al., 2012; MacKenzie-Graham et al., 2012b; Burns et al., 2014; Sun et al., 2015; Meyer et al., 2020
Microglial activation	Girolamo et al., 2011; Mangiardi et al., 2011; Errede et al., 2012; Burns et al., 2014; Gentile et al., 2015; Potter et al., 2016; Meyer et al., 2020
Neuronal loss	Mangiardi et al., 2011; MacKenzie-Graham et al., 2012b; Burns et al., 2014; Stanojlovic et al., 2016; Itoh et al., 2017; Hamilton et al., 2019; Meyer et al., 2020
Synaptic loss	Mangiardi et al., 2011; MacKenzie-Graham et al., 2012b; Burns et al., 2014; Itoh et al., 2017; Meyer et al., 2020

## Hippocampus

Hippocampal involvement in depression and in learning and memory dysfunction has been well documented in MS (Rocca et al., 2018). Hippocampal atrophy has been demonstrated in patients with MS, with worse atrophy in SPMS patients than in RRMS patients (Sicotte et al., 2008). Interneuron loss (parvalbumin) in the CA1 region and hippocampal atrophy was shown in chronic EAE (Ziehn et al., 2010; Hamilton et al., 2019). Interestingly, mice with chronic MOG 35–55 EAE exhibited a deficit in hippocampal-dependent spatial learning and memory in the Barnes maze (Ziehn et al., 2010), as well as altered excitatory synaptic transmission and paired-pulse facilitation (Ziehn et al., 2012a). Neuropathology revealed demyelination in the hippocampus in MS (Vercellino et al., 2005; Geurts et al., 2007; Dutta et al., 2011, 2013), a pathology also observed in chronic EAE (Ziehn et al., 2012b; Bellizzi et al., 2016). Hippocampal demyelination is often associated with synaptic reduction (Dutta et al., 2011; Michailidou et al., 2015) and changes in synaptic composition and transmission in MS (Dutta et al., 2011, 2013). Hippocampal demyelination, synaptic loss and changes in synaptic transmission have also been demonstrated in chronic EAE (Ziehn et al., 2012a,b). Microglial activation has been shown in hippocampal tissues in both humans with MS (Geurts et al., 2007; Dutta et al., 2011) and mice with chronic EAE (Aharoni et al., 2005; Ziehn et al., 2012b; Hammond et al., 2020). Table 2 summarizes hippocampal pathologies in MOG 35–55 EAE.

**TABLE 2:** Pathologies in the hippocampus in EAE.

Pathology	References
**Hippocampus**	
Atrophy	Ziehn et al., 2010, 2012a,b; Hamilton et al., 2019
Demyelination	Ziehn et al., 2010, 2012a,b; Mangiardi et al., 2011; Bellizzi et al., 2016; Hamilton et al., 2019
Microglial activation	Aharoni et al., 2005; Ziehn et al., 2010, 2012a,b; Di Filippo et al., 2013, 2016; Nistico et al., 2013; Gentile et al., 2015; Bellizzi et al., 2016; Planche et al., 2017; Hammond et al., 2020; Bourel et al., 2021
Neuronal loss	Ziehn et al., 2010
Synaptic loss	Ziehn et al., 2010, 2012a,b; Bellizzi et al., 2016; Hammond et al., 2020; Bourel et al., 2021
Synaptic transmission	Ziehn et al., 2012a,b; Di Filippo et al., 2013, 2016, 2021; Nistico et al., 2013; Novkovic et al., 2015; Planche et al., 2017; Kammel et al., 2018

## Thalamus

The thalamus has extensive cortical and sub-cortical connections. The involvement of the thalamus in MS is well documented, with early reports demonstrating thalamic atrophy in MS patients (Cifelli et al., 2002; Wylezinska et al., 2003) and later work demonstrating that thalamic atrophy is detectable early in disease (Brex et al., 2000; Calabrese et al., 2011; Zivadinov et al., 2013; Voskuhl et al., 2020). In fact, thalamic atrophy is a strong predictor for cognitive decline in MS patients (Houtchens et al., 2007; Batista et al., 2012; Schoonheim et al., 2012; Benedict et al., 2013) and is correlated with the accumulation of disability in patients with MS (Rocca et al., 2010; Magon et al., 2020). Early thalamic atrophy has also been reported in chronic EAE (Meyer et al., 2020). Demyelination was reported in postmortem thalami of MS patients (Vercellino et al., 2005), with a later study validating that finding and reporting microglial activation (Vercellino et al., 2009). In parallel, demyelination, astrocyte and microglial activation have been shown in the thalamus in chronic EAE (Aharoni et al., 2005, 2013; Wagenknecht et al., 2016; Werneburg et al., 2020). Neuronal loss in the thalamus in MS has been shown repeatedly (Cifelli et al., 2002; Vercellino et al., 2005, 2009) and neuronal loss (NeuN) in the ventral posterolateral nucleus was observed in chronic EAE (Wagenknecht et al., 2016). Interestingly, synaptic loss in the thalamus in both MS and EAE was shown in the same study (Werneburg et al., 2020). Despite a very large literature on thalamic involvement in MS, there are relatively fewer reports on the thalamus compared to other substructures in EAE. That said, those that do documented thalamic atrophy, demyelination, microglial activation, synaptic and neuronal loss in chronic EAE, as shown in Table 3.

**TABLE 3:** Pathologies in the thalamus in EAE.

Pathology	References
**Thalamus**	
Astrocyte activation	Wagenknecht et al., 2016; Werneburg et al., 2020
Atrophy	Meyer et al., 2020
Demyelination	Aharoni et al., 2013
Microglial activation	Aharoni et al., 2005; Wagenknecht et al., 2016; Werneburg et al., 2020
Neuronal loss	Wagenknecht et al., 2016
Synaptic loss	Werneburg et al., 2020

## Striatum

The striatum comprises the caudate, putamen, and nucleus accumbens, structures associated with both sensory and motor function, as well as cognition and emotion processing. Early studies demonstrated caudate atrophy in MS patients compared to healthy controls (Bermel et al., 2002, 2003), a finding that was replicated in chronic EAE (Hamilton et al., 2019; Meyer et al., 2020). In a comprehensive study of deep GM pathology in postmortem MS patients, demyelination, microglial activation, and neuronal loss were observed in the striatum (Vercellino et al., 2009) and another study by the same group also observed synaptic loss (Vercellino et al., 2007). Astrocyte and microglia activation and synaptic loss have all been shown in chronic EAE (Aharoni et al., 2005; Centonze et al., 2009; Ruffini et al., 2013). Furthermore, alternations in synaptic transmission in the striatum of mice with EAE has been demonstrated many times (Centonze et al., 2007; Gentile et al., 2015; Kammel et al., 2018). Striatal pathologies in MOG 35–55 EAE are listed in Table 4.

**TABLE 4:** Pathologies in the striatum in EAE.

Pathology	References
**Striatum**	
Astrocyte activation	Aharoni et al., 2005; Grasselli et al., 2013; Ruffini et al., 2013; Ravelli et al., 2019
Atrophy	Hamilton et al., 2019; Meyer et al., 2020
Microglial activation	Aharoni et al., 2005; Centonze et al., 2009; Parodi et al., 2015; Gentile et al., 2016
Synaptic loss	Centonze et al., 2009; Ruffini et al., 2013
Synaptic transmission	Centonze et al., 2007, 2009; Rossi et al., 2011; Grasselli et al., 2013; Ruffini et al., 2013; Gentile et al., 2015, 2016; Parodi et al., 2015; Kammel et al., 2018

## Cerebellum

The cerebellum plays a critical role in coordination and balance. Cerebellar deficits have been shown in MS patients (Weinshenker et al., 1996; Alusi et al., 2001) and cerebellar dysfunction was correlated with cerebellar atrophy (Edwards et al., 1999; Liu et al., 1999). Cerebellar atrophy has been shown multiple times in chronic EAE (MacKenzie-Graham et al., 2006, 2009; Hamilton et al., 2019). Interestingly, cerebellar atrophy has been shown to be very strongly correlated with cumulative disease score in chronic EAE (MacKenzie-Graham et al., 2012a), indicating an intimate relationship between cerebellar volume and walking disability. Demyelination in both the cerebellar cortex and cerebellar WM, combined with Purkinje cell loss, was described in postmortem MS patients (Kutzelnigg et al., 2007; Redondo et al., 2015). Similarly, cerebellar cortex and cerebellar WM demyelination, as well as Purkinje cell loss (calbindin and parvalbumin), has been reported in chronic EAE (MacKenzie-Graham et al., 2009, 2012a; Stanojlovic et al., 2016). Other studies have validated cerebellar demyelination and observed extensive microglial activation in postmortem MS tissue (Kemp et al., 2016), as well as astrocyte activation (Albert et al., 2017). Astrocyte and microglia activation have also been shown in chronic EAE (MacKenzie-Graham et al., 2012a; Mandolesi et al., 2013, 2017). Synaptic loss (Albert et al., 2017), as well as axonal damage and transection, have been reported in cerebellum of MS patients (Redondo et al., 2015), and similarly axonal damage (NF200) and synaptic loss have been shown in chronic EAE (MacKenzie-Graham et al., 2009). We have summarized these findings in the cerebellum in chronic EAE in Table 5.

**TABLE 5:** Pathologies in the cerebellum in EAE.

Pathology	References
**Cerebellum**	
Astrocyte activation	Mandolesi et al., 2013
Atrophy	MacKenzie-Graham et al., 2006, 2009, 2012a,2012b; Itoh et al., 2017; Hamilton et al., 2019; Meyer et al., 2020
Axonal damage	MacKenzie-Graham et al., 2009
Demyelination	MacKenzie-Graham et al., 2009, 2012a; Mangiardi et al., 2011; Itoh et al., 2017; Hamilton et al., 2019
Microglial activation	MacKenzie-Graham et al., 2012a; Mandolesi et al., 2013, 2017
Neuronal loss	MacKenzie-Graham et al., 2009, 2012a; Stanojlovic et al., 2016; Itoh et al., 2017; Hamilton et al., 2019
Synaptic loss	MacKenzie-Graham et al., 2009; Itoh et al., 2017
Synaptic transmission	Mangiardi et al., 2011; Mandolesi et al., 2013, 2017

## Retina/optic nerve

Optic neuritis is a common first presentation of MS (Ransohoff et al., 2015) and MS patients can have a persistent reduction in vision after optic neuritis (Cole et al., 2000). Atrophy of the optic nerve has been observed in MS patients with optic neuritis (Hickman et al., 2001; Harrigan et al., 2017) and also in mice with chronic EAE (Hamilton et al., 2019). Demyelination of the optic nerve has been well documented for many years in both MS (Toussaint et al., 1983; Mogensen, 1990) and EAE (Sun et al., 2007; Horstmann et al., 2013; Tassoni et al., 2019). A comprehensive analysis of retinal and optic nerve head neuropathology in MS patients by Green et al. (2010) demonstrated astrocyte and microglia activation, axonal damage and loss, and the loss of retinal ganglion cells. Similarly, astrocyte and microglia activation, axonal damage and loss (APP, NF200, SMI-31, and SMI-312), and the loss of retinal ganglion cells (Brn3a) have also been seen in chronic EAE (Larabee et al., 2016; Jin et al., 2019; Tassoni et al., 2019). Interestingly, retinal nerve fiber layer (RNFL) thinning (Tassoni et al., 2019) and ganglion cell complex (GCC) thinning (Nishioka et al., 2019) has been observed using optical coherence tomograph (OCT), and very strong correlations between GCC thickness and DTI measures of fractional anisotropy and radial diffusivity have also been shown in chronic EAE (Nishioka et al., 2019). Visual acuity was also found to be significantly decreased in mice with chronic EAE (Lin et al., 2014a). Extensive work done in the retina and optic nerve (anterior visual pathway) in chronic EAE is summarized in Table 6.

**TABLE 6:** Pathologies in the retina/optic nerve in EAE.

Pathology	References
**Retina/optic nerve**	
Astrocyte activation	Horstmann et al., 2013, 2016; Jin et al., 2019; Manogaran et al., 2019; Tassoni et al., 2019; Wu et al., 2021; Guo et al., 2022
Atrophy	Hamilton et al., 2019
Axonal damage	Sun et al., 2007; Chaudhary et al., 2011; Lin et al., 2014a,b; Manogaran et al., 2019; Nishioka et al., 2019; Tassoni et al., 2019; Marenna et al., 2020; Yang et al., 2021
Demyelination	Sun et al., 2007; Chaudhary et al., 2011; Horstmann et al., 2013, 2016; Lin et al., 2014a,b; Larabee et al., 2016; Manogaran et al., 2019; Nishioka et al., 2019; Tassoni et al., 2019; Marenna et al., 2020; Wu et al., 2021; Yang et al., 2021; Guo et al., 2022
Immune infiltration	Chaudhary et al., 2011; Horstmann et al., 2013, 2016; Lin et al., 2014a,b; Larabee et al., 2016; Jin et al., 2019; Manogaran et al., 2019; Wu et al., 2021
Microglia activation	Sun et al., 2007; Horstmann et al., 2013; Horstmann et al., 2016; Larabee et al., 2016; Jin et al., 2019; Manogaran et al., 2019; Marenna et al., 2020; Wu et al., 2021; Guo et al., 2022
Neuronal loss	Horstmann et al., 2013, 2016; Larabee et al., 2016; Jin et al., 2019; Manogaran et al., 2019; Nishioka et al., 2019; Tassoni et al., 2019; Wu et al., 2021; Guo et al., 2022
Synaptic loss	Jin et al., 2019

## Corpus callosum

Early MS studies using MRI showed not only corpus callosum atrophy, but that callosal atrophy was associated with brain atrophy and the duration and severity of clinical symptoms (Dietemann et al., 1988), findings that have been reproduced many times (Pelletier et al., 2001; Granberg et al., 2015). Callosal atrophy has also been shown recently in chronic EAE (Hamilton et al., 2019). Callosal demyelination has been shown by histological staining (Barnard and Triggs, 1974) and diffusion MRI (Ozturk et al., 2010) in MS. Similarly, studies using chronic EAE have also shown demyelination in the corpus callosum (Aharoni et al., 2013; Moore et al., 2013). Postmortem studies in MS demonstrated substantial regional axonal loss in the corpus callosum that correlated with regional lesion load and callosal atrophy (Evangelou et al., 2000a,b). Axonal damage and loss (APP, NF200, and SMI-32) and decreased synaptic transmission have also been shown in the corpus callosum in chronic EAE (Mangiardi et al., 2011).

## Discussion

Progressive GM atrophy is strongly associated with clinical disability, making it an important marker for disease progression in MS (Chard et al., 2002; Pirko et al., 2007; MacKenzie-Graham et al., 2016). Understanding neurodegenerative mechanisms that lead to GM atrophy are thus critical. Inflammation, demyelination, and neurodegeneration are intimately connected in MS. In order to understand neurodegeneration in MS, we need models that exhibit neurodegeneration and atrophy in the GM in the context of inflammation and demyelination in the WM. That model is chronic EAE induced with MOG 35–55 peptide in C57BL/6 mice.

Multiple sclerosis and chronic EAE exhibit many of the same pathologies associated with neurodegeneration such as axonal ovoids and end bulbs, synaptic loss, and even neuronal loss in multiple neuroanatomical structures. Atrophy in the cerebral cortex, hippocampus, thalamus, striatum, and cerebellum occur in both MS and chronic EAE. The presence of these key elements of neurodegeneration in chronic EAE suggest that it is a good model to study neurodegeneration in MS.

Outside of relapses, clinical disability worsening in MS is progressive and does not improve significantly. Clinical disabilities are heterogeneous in MS, affecting walking, vision, cognition, and coordination. Standard MS clinical scales such as the Expanded Disability Status Scale (EDSS) are more sensitive to walking disability than other disabilities. At high scores the EDSS scale becomes insensitive to progression, even in walking disability. Progression of GM atrophy continues even during the time when high EDSS scores have plateaued. Other more sensitive outcome measures are used to evaluate progression of disability in vision and cognition. Clinical disability in the chronic EAE model as measured by standard EAE walking scores also reaches a plateau, without improvement. Progression of GM atrophy on MRI continues in chronic EAE over time during the plateau (MacKenzie-Graham et al., 2012b). Beyond walking, disability in vision and cognition have also been shown in chronic EAE using other more sensitive outcome measures (Ziehn et al., 2010; Lin et al., 2014a; Itoh et al., 2018; Tassoni et al., 2019).

Cuprizone toxicity has been suggested as a model to study demyelination in MS. Short-term cuprizone treatment in the diet induces demyelination without axonal damage and complete remyelination after return to normal diet. Chronic cuprizone treatment causes some axonal damage with only partial remyelination (Crawford et al., 2009; Voskuhl et al., 2019), and is thereby more similar to MS. However, the cuprizone model does not entail systemic autoimmunity nor immune cell infiltration into the CNS. Thus, while the cuprizone model is useful to study demyelination and remyelination, it lacks a pathology crucial to MS, namely autoimmunity and infiltrating immune cells. Clearly, chronic demyelination is an important pathology and remyelination is a goal in MS, but inflammation critically affects these processes. It has been shown that the inflammatory microenvironment in MS lesions inhibits the remyelinating capacity of oligodendrocytes in MS and chronic EAE (Kim et al., 2018; Starost et al., 2020). Thus, the presence of inflammation is an essential element in a model that seeks to assess treatments aiming to induce remyelination in MS.

Lysolecithin injections into the CNS induce localized demyelination. The injection is often followed by immune infiltration at the site that appears to be beneficial for remyelination (Kotter et al., 2001; Bieber et al., 2003; Miron et al., 2013). However, there are two important aspects related to neuroaxonal degeneration that warrant consideration in the lysolecithin model. First, axonal damage is not a feature of the lysolecithin model, while axonal damage is known to be an important characteristic of MS (Trapp et al., 1998, 1999). This is key since axonal integrity affects remyelination. Second, MS lesions do not occur in an otherwise healthy CNS. Instead, MS lesions occur in CNS tissues characterized by multifocal pre-existing WM lesions as well as GM pathology (Trapp et al., 1998, 1999; Peterson et al., 2001; Stadelmann et al., 2011; Friese et al., 2014; Mock et al., 2021). Wallerian degeneration from remote lesions could negatively impact remyelination of a new lesion. Even in clinically isolated syndrome (CIS), MRI often demonstrates multiple lesions and early GM atrophy (Brex et al., 2000; Dalton et al., 2004). Thus, identification of remyelination strategies in the lysolecithin model would be most appropriate for determining how to remyelinate a single demyelinating lesion in an otherwise normal CNS, but less aligned with modeling remyelination in a multifocal disease with ongoing pathologies over time.

No animal model recapitulates all aspects of human disease. So too, chronic EAE is not MS and it also has limitations. Ultimately, the MS model chosen depends on the question asked. B-cell involvement in MS is well documented (Meinl et al., 2006; von Budingen et al., 2011) and MOG 35–55 EAE does not require B-cells to induce disease (Hjelmström et al., 1998). Further, MOG 35–55 peptide does not efficiently activate B cells nor promote MOG-specific antibody production (Weber et al., 2010), both hallmarks of MS. That said, EAE induction with recombinant human MOG induces a B cell–dependent disease (Oliver et al., 2003; Marta et al., 2005) suggesting it as a model to study the role of B-cells in MS.

Cortical lesions are characteristic in MS (Geurts et al., 2005; Filippi et al., 2010; Lucchinetti et al., 2011). Although small demyelinating lesions have been reported in MOG 35–55 induced chronic EAE (Mangiardi et al., 2011; MacKenzie-Graham et al., 2012b), there are not large demyelinating cortical lesions. Recent work has produced large demyelinating lesions using a modified MOG 35–55 EAE induction protocol which entailed additional intrathecal injections of TNF-alpha and IFN-gamma into the primary somatosensory cortex (Orefice et al., 2020). This more complex induction paradigm also exhibits neuronal loss, making it appealing for studying large demyelinating cortical lesions in MS.

Experimental autoimmune encephalomyelitis induced by immunization of non-obese diabetic (NOD) mice with MOG 35–55 peptide has been described as having relapses with worsening disability (Ignatius Arokia Doss et al., 2015). The NOD EAE model exhibits gadolinium-enhancing lesions as well as WM damage as detected by DTI (Levy et al., 2010; Levy Barazany et al., 2014). Neuropathology includes WM inflammation, demyelination, and glial activation. Notably however, GM atrophy and GM neuropathologies are important features of disability progression in MS, and these have not been described in the NOD model like they have been in chronic EAE in C57BL/6 mice (Tables 1–6). Also, the vast array of genetically modified (transgenic and gene-targeted knock-out) mice that exist on the C57BL/6 background serve as tools which are more readily amenable for use in addressing mechanistic questions in the C57BL/6 EAE model than in the NOD EAE model.

In summary, we have reviewed neuropathologies in MS and chronic EAE in C57BL/6 mice focusing on various neuroanatomical structures. Although originally developed as a WM inflammatory model of MS, chronic EAE also exhibits neurodegeneration in several key GM structures, indeed those affected in MS. The combination of inflammation, demyelination, and neuroaxonal degeneration that characterizes chronic EAE in C57BL/6 mice makes it an excellent model for the development of new therapies targeting CNS cells to halt and repair disability progression in MS.

## Author contributions

Both authors wrote, read, and approved the final manuscript.
